# Chronic conditions and multimorbidity among West African migrants in greater Barcelona, Spain

**DOI:** 10.3389/fpubh.2023.1142672

**Published:** 2023-07-19

**Authors:** Marina J. MacKinnon, Camila A. Picchio, Daniel K. Nomah, Ariadna Rando Segura, Lena van Selm, Emma Fernández, Maria Buti, Sabela Lens, Xavier Forns, Sergio Rodriguez-Tajes, Javier Pamplona, Carmen Lopez, Francisco Rodriguez-Frías, Jeffrey V. Lazarus

**Affiliations:** ^1^Barcelona Institute for Global Health (ISGlobal), Hospital Clínic, University of Barcelona, Barcelona, Spain; ^2^Department of Health, Center for Epidemiological Studies on Sexually Transmitted Infections and HIV/AIDS in Catalonia (CEEISCAT), Generalitat of Catalonia, Badalona, Spain; ^3^Liver Pathology Unit, Biochemistry and Microbiology Service, Hospital Universitari Vall d'Hebron, Barcelona, Spain; ^4^CIBER Hepatic and Digestive Diseases (CIBERehd), Instituto Carlos III, Madrid, Spain; ^5^Liver Unit, Hospital Universitari Vall d'Hebron, Barcelona, Spain; ^6^Liver Unit, Hospital Clínic de Barcelona, IDIBAPS, University of Barcelona, Barcelona, Spain; ^7^Department of Digestive Diseases, Hospital de Santa Caterina, Girona, Spain; ^8^Department of Digestive Diseases, Hospital Trueta, Girona, Spain; ^9^City University of New York Graduate School of Public Health (CUNY SPH), New York, NY, United States

**Keywords:** migrants, hepatitis B virus, metabolic risk factors, multimorbidity, non-communicable chronic disease (NCD)

## Abstract

**Objectives:**

This study aimed to report the prevalence and identify potential risk factors of chronic conditions among West African migrants living in the greater Barcelona area, Spain, and explore the relationship between years of residence in Spain and chronic disease burden.

**Methods:**

This cross-sectional study included 436 adult African migrants who participated in a community-based hepatitis B virus (HBV) screening and vaccination program (HBV-COMSAVA) in the greater Barcelona area from 21 November 2020 to 22 January 2022. Data were analyzed using standard descriptive statistics and bivariable and multivariable logistic regression.

**Results:**

HBV, non-communicable diseases (NCDs) and metabolic risk factors, and multimorbidity prevalence were 9.17, 20.87, and 4.13%, respectively. Being male or having been previously tested for HBV were associated with higher odds of HBV positivity. Associated risk factors for NCDs and metabolic risk factors included living in Spain for >5 years, being female, and being aged ≥50 years.

**Conclusion:**

The high prevalence of chronic conditions in migrant populations supports a need for early detection strategies and tailored public health interventions that aim to reduce the disease burden imposed on migrants and on health systems in host countries.

## 1. Introduction

As of January 2021, migrants made up about 5.3% of the European Union's (EU) total population with an estimated 23.7 million non-EU citizens living in the EU (1). Spain has the second highest number of resident non-nationals in the EU, with migrants accounting for over 10% (5.4 million people) of Spain's population, and the majority are non-EU citizens (1). Over the past decade, there has been a considerable increase in migration to Europe from countries in sub-Saharan Africa (2). Spain is host to around 1 million African migrants (3).

Host countries with large numbers of migrants must be prepared for the health needs of their incoming populations. Some migrants are disproportionately affected by certain infectious diseases due to a high prevalence of the infection in their country of birth (4, 5). Notably, migrant populations living in the EU/European Economic Area (EEA) who are from countries where hepatitis B virus (HBV) infection is highly endemic, such as in sub-Saharan Africa, are disproportionately affected by HBV (6). While only making up about 5% of the EU's population, migrants from countries where HBV is endemic account for an estimated 25% of all chronic HBV cases in the region (7).

Approximately 257–296 million people are living with chronic HBV infection globally (8, 9). About 1.4 million deaths per year are viral hepatitis related and, of these, nearly half of these deaths can be attributed to HBV alone (6). The prevalence of HBV surface antigen (HBsAg) in the general Spanish population is estimated to be 0.66% (10). However, among migrants who were born in intermediate (HBsAg 2–7%) and high endemic countries (HBsAg ≥8%) and live in the EU/EEA, the prevalence of chronic HBV infection is ~6% (4). Based on these data, it is estimated that migrants from endemic countries account for almost 40% of all chronic HBV infected cases in Spain (10). One study found that in Spain, African migrants accounted for the highest proportion of HBV prevalence in the population (11). In addition, late presentation to viral hepatitis specialist care is frequent in Spain and 35% of HBV patients who present late to care are foreign-born (12). HBV poses a significant public health burden that can be reduced with vaccination, early screening, and treatment (13, 14).

Multimorbidity, the coexistence of two or more chronic conditions in the same individual, is also an increasingly important public health concern (15, 16), especially due to aging populations and the increase in non-communicable diseases (NCDs) globally (17). In general, NCDs account for 71% of annual global deaths, or 41 million mortalities (18, 19). Chronic conditions, including not only NCDs, but also metabolic risk factors such as obesity, hypertension, and high cholesterol, all contribute to the increase in multimorbidity (15). Furthermore, the burden of multimorbidity is greater in vulnerable populations such as migrants, thus worsening health inequalities (15). The estimated prevalence of multimorbidity among African migrants in Aragon, Spain, in 2010 was 11.7% (20).

Migrants may be at increased risk for overall poor health due to structural and sociocultural barriers which hinder their ability to access and navigate the health system in their host country (21). The issue of underutilization of health care services highlights the need for disease screening in migrant populations and the need for accurate and up-to-date epidemiological data for these populations to inform and implement appropriate public health measures. Data are especially lacking on the prevalence and associated risk factors of chronic conditions for African migrants living in Spain, which are key to the design of relevant targeted health interventions, from which this population could benefit. This study aimed to report the prevalence and identify potential risk factors of chronic conditions among African migrants living in Spain and explore the relationship between years of residence in Spain and chronic disease burden.

## 2. Methods

### 2.1. Study design

This research was part of the larger cross-sectional study and community-based hepatitis B screening and vaccination program (HBV-COMSAVA study). This intervention used point-of-care diagnostics in community and faith-based settings in the greater Barcelona area, Catalonia, Spain to identify West African migrants (adults ≥18 years), mainly from Ghana and Senegal, living with HBV. The main objective of the HBV-COMSAVA study was to link migrants to specialist care (22).

### 2.2. Study population

Convenience sampling was used and participants were recruited from study sites provided by community champions and the Association of Ghanaians in Catalonia (ASGC), The Coordinated Association of Senegalese in Catalonia (CASC), and The Cultural Association of African Muslims in Barcelona (CAAMB). These study sites were mainly located in places of worship and community spaces. Study sites that were affiliated with religious spaces (i.e., churches, mosques) allowed researchers to use ongoing religious services/gatherings in order to perform the intervention, posing a significant advantage for the implementation of an intervention directed at a difficult-to-reach population. Community leaders and champions, who were contacted by the study's community coordinators, made announcements to recruit participants, and those present on intervention days were invited to participate. Interventions took place every 2–4 weeks at different study sites throughout the greater Barcelona area. The inclusion criteria for participation in the study were being 18 years or older, providing written informed consent, and being in possession of a regional public health system health card (CatSalut card) or having received one through an expedited process for this project. Exclusion criteria included being unable to understand Spanish, Catalan, English or any other language used by the health care providers, and/or currently being treated for HBV infection.

### 2.3. Data collection

After meeting the inclusion criteria and providing informed consent, participants completed an epidemiological survey consisting of questions regarding sociodemographic variables, HBV-associated risk factors, vaccination status, HBV vaccination criteria, and the presence of any other chronic or acute medical conditions. Once completed, participants were offered a rapid diagnostic test (RDT) (DETERMINE^®^ HBsAg 2, Abbott Laboratories) to screen for HBsAg. This RDT meets the EU regulatory requirements and the WHO International HBsAg Standard with an analytical sensitivity of 0.1 IU/mL (23). A blood sample (140 μl per spot for total of 3 spots) was collected intravenously and spotted onto a plasma separation card (cobas^®^ plasma separation card (PSC), Roche Diagnostics) for analysis in the laboratory. HBV viral load (HBV-DNA) and hepatitis D virus antibodies (anti-HDV) were analyzed among those who were HBsAg+ and HBV core antibodies (anti-HBc) to test for past resolved HBV infection were examined among those HBsAg–. PSCs were transported to Vall d'Hebron University Hospital Laboratory (Barcelona) for analysis 1–7 days after collection. Participants received the results of the RDT on the same day of the intervention. Those who were HBsAg+ were offered a referral to a specialist during their first visit. Laboratory results were communicated during follow-up visits at intervention sites and participants received either post-test-counseling or were offered vaccination against HBV depending on their laboratory results. All patient-reported data collected from the epidemiological survey, human-read RDT results, and laboratory reports from participant blood samples were recorded in an excel database using anonymous patient ID codes. Double entry was used to ensure the accuracy of values.

### 2.4. Variables

All variables were based on self-reported survey data except HBV infection status results, which were collected based on the human-read RDT result.

Sex was reported as female or male. Age was calculated by the reported year of birth. Age was recategorized into four groups: 18–29 years, 30–39 years, 40–49 years, and ≥50 years. Country of origin was reported by country name and recategorized for analysis into three groups: Ghana, Senegal, and Other African countries (Other). Number of children was reported as a continuous variable and recategorized for analysis into 3 groups: 0–2 children, 3–5 children, and ≥6 children. The survey question for the number of children was added on March 7, 2021, so data for this variable were only recorded for the last 281 participants.

Education status of participants was recorded as the highest level of education they had completed: no education, primary school completed, secondary school completed, university bachelor's degree, vocational/trade school, or university master's degree or higher. For analysis, education was recategorized as no schooling, primary completed, secondary completed, bachelor's degree or higher, and vocational/trade school. For employment, participants were asked to choose one of the following categories: full-time work (40 h per week), part-time work (<40 h per week), recently unemployed (<3 months), unemployed (3–12 months), unemployed (>12 months), autonomous worker, student, or other. For analysis, employment was recategorized as unemployed (non-student), employed, or student/other.

The number of years in Spain was calculated by participants' reported year of arrival to Spain. For analysis, number of years in Spain was categorized as 5 years or less and >5 years to distinguish between newly arrived and non-newly arrived migrants (4).

Body mass index (BMI) (kg/m^2^) was calculated as a continuous variable based on self-reported height and weight data. For analysis, BMI was recategorized as underweight (<18.5 kg/m^2^), normal weight (18.5–24.9 kg/m^2^), overweight (25.0–29.9 kg/m^2^), and obese (≥30.0 kg/m^2^). The survey questions for height and weight were added March 21, 2021, so data for BMI calculation were only recorded for the last 261 participants. Data for HBV infection status were based on the RDT result, and when applicable, laboratory confirmation. Participants who tested positive for the presence of HBsAg were categorized as having an active HBV infection. Participants who tested negative for HBsAg were categorized as not having a current HBV infection. Participants were asked if they had any other illnesses to report and this information was used to determine if participants had any other chronic condition besides HBV. Chronic conditions were then categorized as a “NCD or metabolic risk factor” (including obesity, hypertension, and high cholesterol) or “other chronic condition.” HIV, HCV, or an unspecified STI were categorized as other chronic conditions unless otherwise specified by participant.

Participants who reported unclear or unspecific ailments, such as “itchy eyes,” were reclassified as having “no other condition” due to lack of clarity or connection to a specific condition. Participants who reported diseases for which they were already recovered were also reclassified as having no other condition. Participants who reported general symptoms such as back pain without a specific diagnosis were reclassified as having “other health problem.” Multimorbidity was categorized based on the coexistence of two or more chronic conditions and included HBV, NCDs and metabolic risk factors, HCV, unspecified STI, and/or HIV and was recorded dichotomously as yes or no. Absence of multimorbidity was defined by the presence of only one or no chronic conditions.

Travel to Africa was recorded as yes or no based on participant's response to having traveled to the African continent in the last year or planning to travel in the next 12 months. Familial HBV diagnosis, maternal HBV diagnosis, and household HBV diagnosis were all recorded as yes, no, or not sure. HBV vaccination status and previous HBV testing were based on participants' answers to ever having been vaccinated for HBV or ever having been tested for HBV, respectively, and were also recorded as yes, no, or not sure. Ever being incarcerated, having tattoos or scarring, and ever having a surgery were recorded as yes or no.

### 2.5. Data adjustments

Of the 444 individuals surveyed as part of the HBV-COMSAVA intervention, eight participants were excluded from this analysis due to already being in treatment or care for HBV or having an inadequate blood sample for the RDT and/or laboratory results, resulting in a sample size of 436 participants (see Supplementary Figure 1).

### 2.6. Statistical procedures

The raw data were analyzed with StataCorp statistical software: Release 17 (24). Normal distributions of the quantitative variables were evaluated using histograms and Shapiro-Wilk Test. Baseline characteristics of participants were described and summarized. Missing values were accepted unless >5% of data were missing for a single variable. Means with standard deviation (SD) were reported for continuous variables with normal distribution while medians with 25 and 75 percentiles were reported for continuous variables with non-normal distributions. Frequencies and percentages were calculated for categorical variables. Proportions with 95% confidence intervals were measured to assess the prevalence of HBV and other chronic conditions. Bar charts were used to graphically represent the prevalence of HBV and other chronic conditions across different age groups, sex, and years since arrival in Spain.

Pearson's chi square test or Fisher's exact test where appropriate were used to compare proportions between chronic condition categories for each characteristic. The student's *T*-test was used to compare mean values for continuous variables with normal distribution and Mann Whitney-U test was used to compare means for continuous variables with non-normal distribution. Logistic regression models were applied to determine the associations between HBV, NCDs and metabolic risk factors, multimorbidity and variables of interest (years spent in Spain controlling for age, sex, and educational level if applicable). All variables that had exhibited an association with a *P* value of ≤ 0.1 in the bivariable analysis were also included in the multivariable analysis. A *P* value of < 0.05 was considered statistically significant for all analyses.

## 3. Results

### 3.1. Description of study participants

Participants had a median age of 43 (range 19–78). The median number of years residing in Spain was 14 with the majority (337/436, 77.29%) having resided in Spain for more than 5 years. The majority of the study population was male (263/436, 60.32%), had completed secondary level of education (242/436, 55.50%), was employed (289/436, 66.28%), and had recent and/or planned travel to Africa (255/436, 58.49%). Participants were primarily from Ghana (*n* = 320; 73.39%) and Senegal (*n* = 100; 22.94%). The remaining 16 (3.67%) participants were from Mali (*n* = 6; 1.38%), Cameroon (*n* = 2; 0.46%), Niger (*n* = 2; 0.46%), Gambia (*n* = 2; 0.46%), Nigeria (*n* = 1; 0.23%), Benin (*n* = 1; 0.23%), South Africa (*n* = 1; 0.23%), and Egypt (*n* = 1; 0.23%) (Table 1).

**Table 1:** Participants' characteristics (*N* = 436) overall and sorted by presence of chronic condition(s) (HBV-COMSAVA study, Spain, 2020–2022).

	**Overall (*N* = 436)**	**HBV (*n* = 40)**	**NCD/metabolic risk factor (*n* = 91)**	**Multimorbidity (*n* = 18)**	**No chronic condition (*n* = 182)**
**Sex**, ***n*** **(%)**					
Female	173 (39.68)	10 (25.00)	42 (46.15)	6 (33.33)	59 (32.42)
Male	263 (60.32)	30 (75.00)	49 (53.85)	12 (66.67)	123 (67.58)
**Age in years, median (25** ***–*** **75%)**	**43 (36–49)**	**40 (36–48.5)**	**47 (40–52)**	**49.5 (45–52)**	**42 (32–46)**
**Age groups in years**, ***n*** **(%)**					
18–29	61 (13.99)	4 (10.00)	6 (6.59)	1 (5.56)	32 (17.58)
30–39	89 (20.41)	15 (37.50)	14 (15.38)	2 (11.11)	46 (25.27)
40–49	191 (43.81)	14 (35.00)	38 (41.76)	6 (33.33)	78 (42.86)
≥50	94 (21.56)	7 (17.50)	32 (35.16)	9 (50.00)	26 (14.29)
Missing values	1 (0.23)	0 (0)	1 (1.10)	0 (0)	0 (0)
**Country of origin**, ***n*** **(%)**					
Ghana	320 (73.39)	31 (77.50)	56 (61.54)	12 (66.67)	112 (61.54)
Senegal	100 (22.94)	9 (22.50)	33 (36.26)	6 (33.33)	58 (31.87)
Other	16 (3.67)	0 (0)	2 (2.20)	0 (0)	12 (6.59)
**Years in Spain, median (25** ***–*** **75%)**	**14 (7–18)**	**13.5 (7–17)**	**17 (12–19)**	**17 (15–19)**	**14 (5.5–19)**
**Years in Spain in groups**, ***n*** **(%)**					
<5	92 (21.10)	7 (17.50)	6 (6.59)	1 (5.56)	45 (24.73)
>5	337 (77.29)	31 (77.50)	83 (91.21)	17 (94.44)	135 (74.18)
Missing values	7 (1.61)	2 (5.00)	2 (2.20)	0 (0)	2 (1.10)
**Number of children, median (25** ***–*** **75%)**	**2 (1–3)**	**2 (2–3)**	**3 (1–3)**	**2 (1.5–3)**	**2 (1–3)**
**Number of children in groups**, ***n*** **(%)**					
0–2	135 (48.04)^a^	13 (61.90)^b^	34 (43.59)^c^	7 (43.75)^d^	89 (49.72)^e^
3–5	100 (35.59)^a^	6 (28.57)^b^	35 (44.87)^c^	5 (31.25)^d^	58 (32.40)^e^
>6	5 (1.78)^a^	0 (0)^b^	1 (1.28)^c^	0 (0)^d^	4 (2.23)^e^
Missing values	41 (14.59)^a^	2 (9.52)^b^	8 (10.26)^c^	4 (25.00)^d^	28 (15.64)^e^
**Education**, ***n*** **(%)**					
No schooling	42 (9.63)	5 (12.50)	14 (15.38)	3 (16.67)	21 (11.54)
Primary completed	87 (19.95)	11 (27.50)	22 (24.18)	7 (38.89)	39 (21.43)
Secondary completed	242 (55.50)	19 (47.50)	50 (54.95)	7 (38.89)	97 (53.30)
Bachelor's degree or higher	50 (11.47)	4 (10.00)	3 (3.30)	1 (5.56)	20 (10.99)
Vocational/trade school	15 (3.44)	1 (2.50)	2 (2.20)	0 (0)	5 (2.75)
**Employment**, ***n*** **(%)**					
Unemployed (non-student)	132 (30.28)	11 (27.50)	27 (29.67)	4 (22.22)	55 (30.22)
Employed	289 (66.28)	29 (72.50)	62 (68.13)	12 (66.67)	120 (65.93)
Student/Other	14 (3.21)	0 (0)	2 (2.20)	2 (11.11)	7 (3.85)
Missing values	1 (0.23)	0 (0)	0 (0)	0 (0)	0 (0)
**BMI kg/m** ^2^ **, median (25** ***–*** **75%)**	**26.2 (23.5–29)**	**23.5 (22.1–29.1)**	**29.3 (24.7–30.8)**	**29.7 (26.4–30.1)**	**25.85 (23.6–27.5)**
**BMI categories**, ***n*** **(%)**					
Underweight (< 18.5 kg/m^2^)	3 (1.15)^f^	0 (0)^g^	1 (1.39)^h^	0 (0)^i^	2 (1.19)^j^
Normal weight (18.5–24.9 kg/m^2^)	62 (23.75)^f^	9 (50.00)^g^	13 (18.06)^h^	2 (13.33)^i^	40 (23.81)^j^
Overweight (25.0–29.9 kg/m^2^)	77 (29.50)^f^	3 (16.67)^g^	17 (23.61)^h^	5 (33.33)^i^	56 (33.33)^j^
Obese (≥30.0 kg/m^2^)	21 (8.05)^f^	3 (16.67)^g^	21 (29.17)^h^	6 (40.00)^i^	0 (0)^j^
Missing values	98 (37.55)^f^	3 (16.67)^g^	20 (27.78)^h^	2 (13.33)^i^	70 (41.67)^j^
**HBV related risk factors**, ***n*** **(%)**					
Travel to Africa	255 (58.49)	26 (65.00)	61 (67.03)	14 (77.78)	117 (64.29)
Tattoos or scarring	7 (1.61)	0 (0)	2 (2.20)	1 (5.56)	4 (2.20)
Previous incarceration	10 (2.29)	0 (0)	2 (2.20)	0 (0)	6 (3.30)
Previous surgery	135 (30.96)	15 (37.50)	31 (34.07)	6 (33.33)	49 (26.92)
Familial HBV diagnosis	36 (8.26)	4 (10.00)	7 (7.69)	1 (5.56)	16 (8.79)
Maternal HBV diagnosis	5 (1.15)	2 (5.00)	2 (2.20)	0 (0)	0 (0)
Household HBV diagnosis	16 (3.67)	0 (0)	2 (2.20)	1 (5.56)	8 (4.40)
Previously vaccinated for HBV	46 (10.55)	4 (10.00)	9 (9.89)	1 (5.56)	19 (10.44)
Previously tested for HBV	79 (18.12)	15 (37.50)	18 (19.78)	6 (33.33)	28 (15.38)

Values are medians or percentages calculated at 95% confidence intervals. Number of children and BMI variables exclude the first 156 and 176 participants of the survey sample, respectively, for whom data was not collected. Reported HBV related risk factor variables' values represent number of “yes” responses.

^a^N=281; ^b^N=21; ^c^N=78; ^d^N=16; ^e^ N=179;^f^ N=261^; *g*^ N=18; ^h^N=72; ^i^N=15; ^j^N=168.

124 participants had missing data for one or more of the chronic condition variables and absence of chronic conditions could not be determined.

BMI, body mass index; HBV, hepatitis B virus; NCD, non-communicable disease.

### 3.2. Prevalence of chronic conditions and multimorbidity

**Figure 1:**
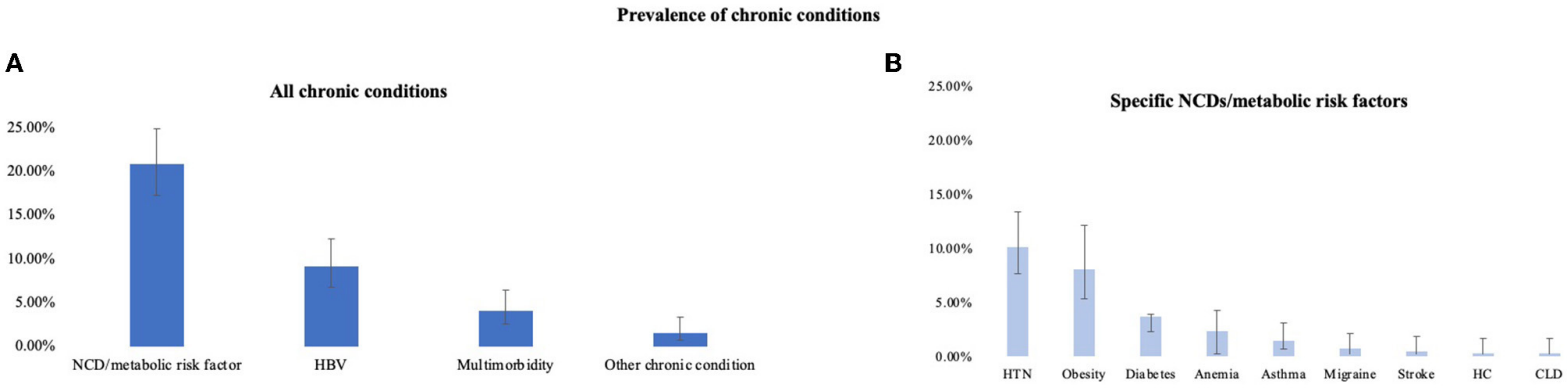
Overall prevalence of chronic conditions **(A)** and specific non-communicable diseases/metabolic risk factors **(B)** with 95% confidence intervals. NCD, non-communicable disease; HBV, hepatitis B virus; HTN, hypertension; HC, high cholesterol; CLD, chronic liver disease. Other chronic conditions included HIV, HCV, and unspecified STI. For obesity variable, *n* = 262. (HBV-COMSAVA study, Spain, 2020–2022).

A summary of the prevalence of chronic conditions is displayed in Figure 1. The overall prevalence of HBV and NCDs/metabolic risk factors in the population was 9.17% (95% CI 6.79–12.28%) and 20.87% (95% CI 17.30–24.95%), respectively. 1.61% (95% CI 0.77–3.34%) of the 436 participants reported having at least one chronic condition other than HBV or an NCD/metabolic risk factor: 1.61% (95% CI 0.77–3.34%) reported having an unspecified STI, 0.46% reported having HIV (95% CI 0.11–1.82%), and 0.23% reported having HCV (95% CI 0.03–1.62%). 4.13% (95% CI 2.61–6.46%) of all participants reported multimorbidity. HBV accounted for one of the two or more conditions among two thirds (*n* = 6) of participants with multimorbidity. The NCDs and metabolic risk factors reported among the 436 participants and their respective prevalence were hypertension 10.09% (95% CI 7.59–13.30%), obesity 8.05% (95% CI 5.29–12.05%), diabetes 3.67% (95% CI 2.26–5.91%), anemia 2.29% (95% CI 1.24–4.22%), asthma 1.38% (95% CI 0.62–3.04%), migraine 0.69% (95% CI 0.22–2.12%), stroke 0.46% (95% CI 0.11–1.82%), high cholesterol 0.23% (95% CI 0.03–1.62%), and chronic liver disease 0.23% (95% CI 0.03–1.62%).

The fully adjusted model for HBV suggests that males have an increased odds (odds ratio [OR] 3.12, 95% CI 1.34–7.23) of having HBV compared to females (Table 2). It was also observed that the odds of having HBV was higher in those who had previously been tested for HBV (OR 4.05, 95% CI 1.78–9.20) compared to those who had not been previously tested for HBV. There were no significant associations between HBV and years residing in Spain. There was also no significant association found between HBV and age or education level. The fully adjusted model for NCDs/metabolic risk factors suggests that people who have lived in Spain for more than 5 years had an increased odds (OR = 3.48; 95% CI: 1.36–8.88) of having an NCD or metabolic risk factor compared to those who have lived in Spain for 5 years or less. Females had an increased odds (OR = 2.09; 95% CI: 1.20–3.65) of having an NCD or metabolic risk factor compared to males and participants aged 50 and above had a significantly higher odds (OR = 3.83; 95% CI: 1.32–11.09) of having an NCD/metabolic risk factor compared to younger participants aged 18–29 years. There were no significant associations in the fully adjusted model for multimorbidity. Detailed results of the bivariable analysis are available in Supplementary Tables 1–3.

**Table 2:** Results of the multivariable logistic regression models for quantifying independent effects of sub-group membership on risk of experiencing chronic conditions (HBV-COMSAVA study, Spain, 2020–2022).

	**Hepatits B virus**			**NCD/metabolic risk factor**			**Multimorbidity**		
	**(*****n*** = **40)**			**(*****n*** = **91)**			**(*****n*** = **18)**		
**Variables included in multivariate models**	**OR**	**95% CI**	***P*** **value**	**OR**	**95% CI**	***P*** **value**	**OR**	**95% CI**	***P*** **value**
**Years in Spain**									
<5 years	1.00 (Ref)			1.00 (Ref)			1.00 (ref)		
>5 years	1.28	0.49–3.30	0.616	3.48	1.36–8.88	**0.009**	2.46	0.30–20.43	0.405
**Sex**									
Male	3.12	1.34–7.23	**0.008**	1.00 (Ref)			1.00 (ref)		
Female	1.00 (Ref)			2.09	1.20–3.65	**0.01**	0.89	0.31–2.53	0.825
**Age**									
18–29	1.00 (Ref)			1.00 (Ref)			1.00 (ref)		
30–39	2.31	0.66–8.07	0.19	0.96	0.32–2.88	0.939	0.99	0.08–11.79	0.997
40–49	0.85	0.25–2.93	0.794	1.69	0.63–4.54	0.3	1.51	0.17–13.52	0.711
≥50	0.96	0.24–3.91	0.954	3.83	1.32–11.09	**0.013**	5.18	0.58–45.93	0.14
**Education**									
No schooling	1.00 (Ref)								
Primary completed	0.95	0.29–3.15	0.937						
Secondary completed	0.52	0.17–1.56	0.242						
Bachelor's degree or higher	0.42	0.10–1.82	0.245						
Vocational/trade school	0.47	0.04–5.03	0.534						
**Previously Tested for HBV**									
No	1.00 (Ref)								
Yes	4.05	1.78–9.20	**0.001**						
Not sure	1.27	0.45–3.64	0.65						

Statistically significant variables (at level α < 0.05) are highlighted in bold. The minimal sufficient adjustment sets applied to this analysis for estimating the total effect of years in Spain on HBV were age, education level, and sex. The minimal sufficient adjustment sets applied to this analysis for estimating the total effect of years in Spain on NCD and metabolic risk factors and on multimorbidity were age, and sex.

CI, confidence interval; OR, odds ratio; NCD, non-communicable disease.

## 4. Discussion

The overall prevalence of HBV found in this study is much higher than the estimated overall HBV prevalence of 0.52% for Catalonia, Spain (25). This is consistent with studies that have found a higher prevalence among migrants, especially those from HBV endemic countries, than in the general European and Spanish population (7, 10). The prevalence of HBV for men was higher than for women in this study. While current literature supports that male sex is a risk factor for HBV prevalence (26), the differences in sex observed in this study could also be explained by the differences in health-seeking behavior among women and men and the exclusion criteria for participation in our study. In general, women have more health-seeking behavior, especially at the primary care level (27). The participants who were excluded for currently receiving HBV treatment were primarily women and may have been more recently tested (data not reported), perhaps due to antenatal screening in Spain. This hypothesis could be explored in future analyses accounting for how recently women and men were last tested for HBV.

Many of the participants in this sample would not be eligible for HBV treatment in their home countries, since in West Africa fewer than 1 in 5 people with chronic HBV are eligible for treatment (28). Ineligibility for HBV treatment or previous loss to follow up may explain why previous HBV testing was found to be associated with HBV infection in this study. Participants may have received, but were currently unaware of, a previous HBV diagnosis and not in regular follow-up or treatment for HBV. Hence, they were not excluded from participating in this study.

Among the most prevalent NCDs/metabolic risk factors in this population were hypertension, obesity, and diabetes. Studies have shown that when migrants from non-Western countries migrate to Western countries, they may be more prone to such diseases in their new obesogenic environment (29, 30). Data also show that migrants coming from Africa to Europe are at a significantly increased risk for developing type 2 diabetes (31). The prevalence of NCDs/metabolic risk factors in this study population resembles the patterns seen in the general migrant population in Spain (20) and Ghanaian migrants in Europe (32): morbidity increases with length of time spent in Spain. These chronic conditions and migrants' years of residence in Spain should be carefully considered when planning screening and treatment interventions for migrant populations.

Age is a known risk factor for NCDs (33) and while participants aged 50 and above in this study population were at a greater risk of having an NCD or metabolic risk factor, prevention and control of chronic conditions must be considered for all age groups and genders. There are marked differences in mortality and morbidity between females and males for different NCDs and metabolic risk factors (34). One hypothesis for the observed increased odds of NCDs/metabolic risk factors among women in this study is that women in this population may have been more likely to have an NCD/metabolic risk factor diagnosis due to their previous health-seeking behavior. Additionally, obesity was accounted for in the definition of NCDs/metabolic risk factors, which is highly prevalent in the Ghanaian population, especially among women (17, 35), and could have also contributed to this observed difference.

The prevalence of multimorbidity (4.13%) was relatively low in the study population compared to other studies that have found a prevalence of up to 50% in African migrants living in Europe (17). However, this study was limited by the fact that multimorbidity could not be determined for participants who had missing data for one or more chronic condition categories. There were no significantly associated risk factors to multimorbidity found in this study likely due to having such a limited sample of participants (*n* = 18) with multimorbidity who had information available. Results did show that out of the 18 participants who had multimorbidity, HBV accounted for one of the two or more chronic conditions among six participants (33%). The fact that these participants already had a diagnosed chronic condition highlights the possible missed opportunity for a previous HBV diagnosis during their health system utilization.

### 4.1. Implications for public health policy

Participants who have been in Spain for more than 5 years had a significantly higher odds of having an NCD/metabolic risk factor compared to newly arrived migrants in this study. Multimorbidity and a high prevalence of HBV and NCDs/metabolic risk factors found in this population suggests that health system utilization is critical upon migrants' initial arrival to Spain. Early detection of chronic conditions can possibly slow progression of disease, reduce mortality, and reduce the burden on health systems that would otherwise result from late detection of chronic conditions. Therefore, interventions should aim to increase linkage to care among newly arrived migrants who may have undiagnosed and/or asymptomatic chronic conditions that will benefit from early intervention and management. When linked to care, especially at the primary care level, it is also important that routine screenings for chronic conditions include screenings for both NCDs/metabolic risk factors and HBV as to make efficient use of migrants' health system utilization, avoid possibly overlooking certain conditions during their visits, and to ensure health and health care equity for this population.

### 4.2. Implications for future research

Future studies, with a larger sample size and study population, could offer more robust data into the burden of chronic conditions on African migrants and their health system utilization in Spain. Public health research should also consider relevant risk factors when designing and evaluating community-based interventions for similar populations.

### 4.3. Limitations

This study had several possible limitations. Firstly, people who were already receiving treatment for HBV were not eligible to participate in the HBV-COMSAVA study. This may have resulted in an underestimation of the prevalence of HBV infection in this population. Since participants were recruited by convenience sampling, this cohort may not be representative and the results of this study may not be generalizable to other points in time or to other West African migrant populations, especially those living outside of the greater Barcelona area. Further studies utilizing random sampling or a cohort study design with longitudinal follow-up rather than a cross-sectional design could provide more robust data on prevalence rates among this population.

Furthermore, the prevalence of NCDs/metabolic risk factors and the prevalence of multimorbidity do not account for potential obesity in 196 participants who were not asked for height and weight information for BMI calculation since these variables were not initially included in the study questionnaire. Additionally, self-reporting of chronic conditions cannot account for patients with undiagnosed conditions. For these reasons, NCDs/metabolic risk factors and multimorbidity prevalence may have been underestimated in this cohort. Also, all survey questions were self-reported and questions about participant histories may have been subject to measurement error bias regarding height and weight data, self-reporting bias, recall bias, and/or social desirability bias.

### 4.4. Conclusion

This study adds to the limited data available regarding the chronic disease burden in African migrants living in Spain. This study found the prevalence of HBV, NCDs/metabolic risk factors, and multimorbidity were 9.17%, 20.87%, and 4.13%, respectively. Being male or having been previously tested for HBV were associated with higher odds of HBV positivity. Being female, being aged 50 or above, and having lived in Spain for more than 5 years were associated risk factors for having an NCD/metabolic risk factor. These results offer valuable information for designing and evaluating community-based interventions. Specifically, these results highlight the need for early detection strategies and tailored public health interventions that aim to increase healthcare utilization among migrants, reduce the burden of chronic conditions in migrant populations, and reduce the burden on health systems in migrants' host countries.

## Data availability statement

The raw data supporting the conclusions of this article will be made available by the authors, without undue reservation.

## Ethics statement

The studies involving human participants were reviewed and approved by Ethical Committee of the Hospital Clínic de Barcelona, Barcelona, Spain (n. HCB/2020/1036). The patients/participants provided their written informed consent to participate in this study.

## Author contributions

CP and JL conceived the study and finalized the study with input from MM. Data collection was carried out by CP, MM, DN, and LS. Laboratory analyses were carried out by AS under the supervision of FR-F. Patients were seen by MB, SL, SR-T, JP, and CL. MM wrote the first draft of the manuscript with input from CP. All authors contributed to subsequent drafts of the manuscript and approved it for submission.
