# Supplementary material for: Chronic conditions and multimorbidity among West African migrants in greater Barcelona, Spain
Source: Front Public Health. 2023 Jul 19;11:1142672. doi: 10.3389/fpubh.2023.1142672 (PMC10394694; doi:10.3389/fpubh.2023.1142672)
Supplement: Supplementary file 1 [file Data_Sheet_1.docx]

Supplementary Material

**Chronic conditions and multimorbidity among West African migrants in greater Barcelona, Spain**

**Marina J MacKinnon^1†,^, Camila A Picchio^1†*^, Daniel K Nomah^2^, Ariadna Rando Segura^3^, Lena van Selm^1^, Emma Fernández^1^, Maria Buti^4,5^, Sabela Lens^4,6^, Xavier Forns^4,6^, Sergio Rodriguez-Tajes^4,6^, Javier Pamplona^7^, Carmen Lopez^8^, Francisco Rodriguez-Frías^3^, Jeffrey V Lazarus^1,9^**

^†^These authors contributed equally to this work and share first authorship

*** Correspondence:** Camila Picchio, camila.picchio@isglobal.org


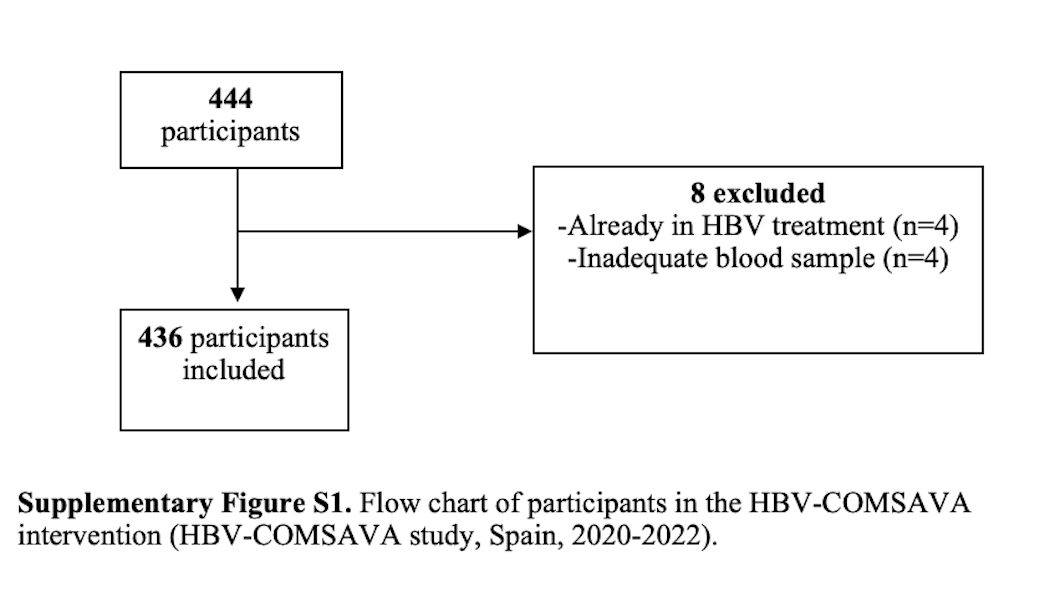


| **Supplementary Table S1.** Results for bivariable analysis of participants’ characteristics according to HBV status (HBV-COMSAVA study, Spain 2020-2022).   \|  \| \| --- \| |  |  |  |  |
| --- | --- | --- | --- | --- | --- |
|  | **HBV =40(9.17)** | **No HBV =396(90.83)** | **P value** |  |
| Sex, n (%) |  |  | **0.046** |  |
| Female | 10(5.78) | 163(94.22) |  |  |
| Male | 30(11.41) | 233(88.59) |  |  |
| Age in years, mean (SD) | 40.75(8.86) | 42.33(10.41) | 0.342 |  |
| Age groups in years, n (%) |  |  | **0.048** |  |
| 18 - 29 | 4(6.56) | 57(93.44) |  |  |
| 30 - 39 | 15(16.85) | 74(83.15) |  |  |
| 40 - 49 | 14(7.33) | 177(92.67) |  |  |
| ≥50 | 7(7.45) | 87(92.55) |  |  |
| Missing values | 0(0) | 1(100.00) |  |  |
| Country of origin, n (%) |  |  | 0.61 |  |
| Ghana | 31(9.69) | 289(90.31) |  |  |
| Senegal | 9(9.00) | 91(91.00) |  |  |
| Other | 0(0) | 16(100.00) |  |  |
| Years in Spain, mean (SD) | 12.66(5.62) | 13.39(7.02) | 0.443 |  |
| Years in Spain in groups, n (%) |  |  | 0.634 |  |
| <5 | 7(7.61) | 85(92.39) |  |  |
| >5 | 31(9.17) | 306(90.53) |  |  |
| Missing values | 2(28.57) | 5(71.43) |  |  |
| Number of children, mean (SD) | 2(1.25) | 2.3(1.63) | 0.567 |  |
| Number of children in groups, n (%) |  |  | 0.568 |  |
|  |  |  |  |  |
| 0-2 | 13(9.63) ^a^ | 122(90.37) ^a^ |  |  |
| 3-5 | 6(6.00) ^a^ | 94(94.00) ^a^ |  |  |
| >6 | 0(0) ^a^ | 5(100.00) ^a^ |  |  |
| Missing values | 2(4.88) ^a^ | 39(95.12) ^a^ |  |  |
| Education, n (%) |  |  | 0.648 |  |
| No schooling | 5(11.90) | 37(88.10) |  |  |
| Primary completed | 11(12.64) | 76(87.36) |  |  |
| Secondary completed | 19(7.85) | 223(92.15) |  |  |
| Bachelor’s degree or higher | 4(8.00) | 46(92.00) |  |  |
| Vocational/trade school | 1(6.67) | 14(93.33) |  |  |
| Employment, n (%) |  |  | 0.591 |  |
| Unemployed (non-student) | 11(8.33) | 121(91.67) |  |  |
| Employed | 29(10.03) | 260(89.97) |  |  |
| Student/Other | 0(0) | 14(100.00) |  |  |
| Missing values | 0(0) | 1(100.00) |  |  |
| Note: Continued on next page.  HBV Related Risk Factors, n (%) | Note: Continued on next page. | Note: Continued on next page. | Note: Continued on next page. | Note: Continued on next page. |
| Travel to Africa | 26(10.20) | 229(89.80) | 0.4 |  |
| Tattoos or Scarring | 0(0) | 7(100.00) | 0.272 |  |
| Previous Incarceration | 0(0) | 10(100.00) | 0.609 |  |
| Previous Surgery | 15(11.11) | 120(88.89) | 0.431 |  |
| Familial HBV Diagnosis | 4(11.11) | 32(88.89) | 0.888 |  |
| Maternal HBV Diagnosis | 2(40.00) | 3(60.00) | 0.128 |  |
| Household HBV Diagnosis | 0(0) | 16(100.00) | 0.38 |  |
| Previously Vaccinated for HBV | 4(8.70) | 42(91.30) | 0.427 |  |
| \| Previously Tested for HBV \| \| --- \| |  |  | **0.004** |  |
| No | 20(6.76) | 276(93.24) |  |  |
|  |  |  |  |  |
|  |  |  |  |  |
| *Values are means or percentages calculated at 95% confidence intervals. Number of children variable excludes the first 156 participants of the survey sample. Reported HBV related risk factor variables’ values represent number of “yes” responses unless otherwise specified.*  *^a^N=281.*  *Notes: Statistically significant variables (at level α< 0.05) are highlighted in bold.  HBV, hepatitis B virus.* |  |  |  |  |
|  |  |  |  |  |
|  |  |  |  |  |
|  |  |  |  |  |
|  |  |  |  |  |

**Supplementary Table S2.** Results for bivariable analysis of participants’ characteristics according to NCD/metabolic risk factor status (HBV-COMSAVA study, Spain 2020-2022).

|  | **NCD/metabolic risk factor =91(20.87)** | **No NCD/metabolic risk factor =202(46.33)** | **Missing =143(32.80)** | **P value** |
| --- | --- | --- | --- | --- |
| Sex, n(%) |  |  |  | **0.01** |
| Female | 42(24.28) | 62(35.84) | 69(39.88) |  |
| Male | 49(18.63) | 140(53.23) | 74(28.14) |  |
| Age in years, mean (SD) | 45.96(9.56) | 40.49(9.85) |  | **<0.001** |
| Age groups in years, n (%) |  |  |  | **<0.001** |
| 18 - 29 | 6(9.84) | 33(54.10) | 22(36.07) |  |
| 30 - 39 | 14(15.73) | 53(59.55) | 22(24.72) |  |
| 40 - 49 | 38(19.90) | 89(46.60) | 64(33.51) |  |
| ≥50 | 32(34.04) | 27(28.72) | 35(37.23) |  |
| Missing values | 1(100.00) | 0(0) | 0(0) |  |
| Country of origin, n (%) |  |  |  | 0.385 |
| Ghana | 56(17.50) | 124(38.75) | 140(43.75) |  |
| Senegal | 33(33.00) | 66(66.00) | 1(1.00) |  |
| Other | 2(12.50) | 12(75.00) | 2(12.50) |  |
| Years in Spain, mean (SD) | 16.13(6.54) | 12.87(6.91) |  | **0.002** |
| Years in Spain in groups, n (%) |  |  |  | **0.001** |
| <5 | 6(6.52) | 46(50.00) | 40(43.48) |  |
| >5 | 83(24.63) | 153(45.40) | 101(29.97) |  |
| Missing values | 2(28.57) | 3(42.86) | 2(28.57) |  |
| Number of children, mean (SD) | 2.46(1.52) | 2.19(1.64) |  | 0.156 |
| Number of children in groups, n (%) |  |  |  | 0.227 |
| 0-2 | 34(25.19) ^a^ | 99(73.33) ^a^ | 2(1.48) ^a^ |  |
| 3-5 | 35(35.00) ^a^ | 64(64.00) ^a^ | 1(1.00) ^a^ |  |
| >6 | 1(20.00) ^a^ | 4(80.00) ^a^ | 0(0) ^a^ |  |
| Missing values | 8(19.51) ^a^ | 31(75.61) ^a^ | 2(4.88) ^a^ |  |
| Education, n (%) |  |  |  | 0.202 |
| No schooling | 14(33.33) | 24(57.14) | 4(9.52) |  |
| Primary completed | 22(25.29) | 44(50.57) | 21(24.14) |  |
| Secondary completed | 50(20.66) | 106(43.80) | 86(35.54) |  |
| Bachelor’s degree or higher | 3(6.00) | 23(46.00) | 24(48.00) |  |
| Vocational/trade School | 2(13.33) | 5(33.33) | 8(53.33) |  |
| Employment, n (%) |  |  |  | 0.883 |
| Unemployed (non-student) | 27(20.45) | 61(46.21) | 44(33.33) |  |
| Employed | 62(21.45) | 134(46.37) | 93(32.18) |  |
| Student/Other | 2(14.29) | 7(50.00) | 5(35.71) |  |
| \| Missing values \| \| --- \| | 0(0) | 0(0) | 1(100.00) |  |
| *Values are means or percentages calculated at 95% confidence intervals. Number of children variable excludes the first 156 participants of the survey sample.*  *^a^N=281.*  *Notes: Statistically significant variables (at level α< 0.05) are highlighted in bold.  NCD, non-communicable disease.* |  |  |  |  |
|  |  |  |  |  |
|  |  |  |  |  |
|  |  |  |  |  |
| **Supplementary Table S3.** Results for bivariable analysis of participants’ characteristics according to multimorbidity status (HBV-COMSAVA study, Spain 2020-2022). |  |  |  |  |
|  |  |  |  |  |

|  | **Multimorbidity =18(4.13)** | | **No Multimorbidity =274(62.84)** | **Missing =144(33.03)** | **P value** |  |
| --- | --- | --- | --- | --- | --- | --- |
| Sex, n(%) |  |  | |  | 0.835 |  |
| Female | 6(3.47) | 98(56.65) | | 69(39.88) |  |  |
| Male | 12(4.56) | 176(66.92) | | 75(28.52) |  |  |
| Age in years, mean (SD) | 48.33(8.38) | 41.76(10.07) | |  | **0.003** |  |
| Age groups in years, n (%) |  |  | |  | **0.027** |  |
| 18 - 29 | 1(1.64) | 38(62.30) | | 22(36.07) |  |  |
| 30 - 39 | 2(2.25) | 65(73.03) | | 22(24.72) |  |  |
| 40 - 49 | 6(3.14) | 120(62.83) | | 65(34.03) |  |  |
| ≥50 | 9(9.57) | 50(53.19) | | 35(37.23) |  |  |
| Missing values | 0(0) | 1(100.00) | | 0(0) |  |  |
| Country of origin, n (%) |  |  | |  | 1 |  |
| Ghana | 12(3.75) | 167(52.19) | | 141(44.06) |  |  |
| Senegal | 6(6.00) | 93(93.00) | | 1(1.00) |  |  |
| Other | 0(0) | 14(87.50) | | 2(12.50) |  |  |
| Years in Spain, mean (SD) | 18(8.20) | 13.59(6.80) | |  | 0.057 |  |
| Years in Spain in groups, n (%) |  |  | |  | 0.212 |  |
| <5 | 1(1.09) | 51(55.43) | | 40(43.48) |  |  |
| >5 | 17(5.04) | 218(64.69) | | 102(30.27) |  |  |
| Missing values | 0(0) | 5(71.43) | | 2(28.57) |  |  |
| Number of children, mean (SD) | 2.33(1.37) | 2.27(1.62) | |  | 0.827 |  |
| Number of children in groups, n (%) |  |  | |  | 1 |  |
|  |  |  |  |  |  |  |
| 0-2 | 7(5.19) ^a^ | 125(92.59) ^a^ | | 3(2.22) ^a^ |  |  |
| 3-5 | 5(5.00) ^a^ | 94(94.00) ^a^ | | 1(1.00) ^a^ |  |  |
| >6 | 0(0) ^a^ | 5(100.00) ^a^ | | 0(0) ^a^ |  |  |
| Missing values | 4(9.76) ^a^ | 35(85.37) ^a^ | | 2(4.88) ^a^ |  |  |
| Education, n (%) |  |  | |  | 0.452 |  |
| No schooling | 3(7.14) | 35(83.33) | | 4(9.52) |  |  |
| Primary completed | 7(8.05) | 59(67.82) | | 21(24.14) |  |  |
| Secondary completed | 7(2.89) | 148(61.16) | | 87(35.95) |  |  |
| Bachelor’s degree or higher | 1(2.00) | 25(50.00) | | 24(48.00) |  |  |
| Vocational/trade school | 0(0) | 7(46.67) | | 8(53.33) |  |  |
| Employment, n (%) |  |  | |  | 0.135 |  |
| Unemployed (non-student) | 4(3.03) | 84(63.64) | | 44(33.33) |  |  |
| Employed | 12(4.15) | 183(63.32) | | 94(32.53) |  |  |
| Student/Other | 2(14.29) | 7(50.00) | | 5(35.71) |  |  |
| Missing values | 0(0) | 0(0) | | 1(100.00) |  |  |
| *Values are means or percentages calculated at 95% confidence intervals. Number of children variable excludes the first 156 participants of the survey sample.*  *^a^N=281.*  *Notes: Statistically significant variables (at level α< 0.05) are highlighted in bold.* |  |  | |  |  |  |
|  |  |  | |  |  |  |
